# Happiness at work: A cross-cultural validation of happiness at work scale

**DOI:** 10.1371/journal.pone.0261617

**Published:** 2022-01-05

**Authors:** Nina Fitriana, Fonny Dameaty Hutagalung, Zainudin Awang, Sumaia Mohammed Zaid

**Affiliations:** 1 Educational Psychology and Counselling Department, Faculty of Education, University of Malaya, Kuala Lumpur, Malaysia; 2 Department of Psychology, Faculty of Psychology, University of Mercu Buana Yogyakarta, Yogyakarta, Indonesia; 3 Faculty of Economics and Management Sciences, University Sultan Zainal Abidin, Terengganu, Malaysia; 4 Department of Psychology, Sana’a University, Sana’a, Yemen; Julius-Maximilians-Universität Würzburg, GERMANY

## Abstract

The idea of Happiness at Work is drawn from psychology and economic studies. It is often considered as a synonym with ‘wellbeing’ and defined as a state characterized by a high level of life satisfaction, a high level of positive emotions, and less negative emotions. This research aims to validate the Happiness at Work scale in the Indonesian context. In this study, the researchers conducted cross-cultural adaptation for the Happiness at Work scale following systematic procedures to produce the Scale of Happiness at Work in the Indonesian language. Afterward, the researchers evaluated the content validity with the help of professional judgment and measured the Content Validity Index at the item level and the scale level. Further, to examine the psychometric properties of the Happiness at Work scale, the researchers administer the questionnaire to a sample of 105 (35 male and 70 female) lecturers to conduct exploratory factor analysis to formulate the new dimensionality of the Happiness at Work scale. The results of Exploratory Factor Analysis indicated that Happiness at Work in the Indonesian context could be measured using four dimensions. To confirm that the extracted dimensions measure a single construct, the researchers administered the produced version to a sample of 370 (147 male and 223 female). Afterward, researchers conducted confirmatory factor analysis to evaluate the validity and reliability of the measurement model. This research found out that the Indonesian version of Happiness at Work measurement is reliable and valid. Thus, this study may contribute to the happiness at work literature of non-western context. In conclusion, the Indonesian-Happiness at Work scale shows robust psychometric properties that can be used for further research.

## Introduction

Happiness is a top priority in people’s lives. The concept of happiness has been widely discussed in western literature for many decades. However, it remains an important topic up to the present. Although the idea of happiness has been conceptually perceived as a general construct that represents a global assessment of one’s life, recently, the concept has been expanded into a new terminology, namely Happiness At Work (HAW) [1, 2]. The idea of HAW has been drawn on happiness from psychology and economic studies. It is often considered as a synonym with well-being and defined as a state characterized by a high level of life satisfaction, a high level of positive emotions, and less negative emotions [3]. Work happiness is also defined as feeling good about work, feeling good about characteristics of the job and feeling right about the entire organization, pleasant judgments or experiences, namely positive feelings, flow at work, moods, and emotions [1].

Fisher [4] provided a model that concentrates on an individual’s happiness at work, mood experience, and pleasant emotions while working in an attempt to integrate the various perspectives on happiness. Many components are incorporated into this model such as mood and positive emotions at work, the satisfaction of judgment at work, and similar attitudes. To be more specific, according to Fisher [1], there are three dimensions of happiness at work. The first dimension is job satisfaction, people’s feelings about the jobs, and the aspects of the job. The second dimension is affective organizational commitment, a condition in which employees admit themselves to a specific organization and its objectives and is characterized by the willingness to stay in the organization longer [5]. The third dimension is engagement, a positive feeling, and satisfaction at work, described by three components, i.e. vigor, dedication, and absorption [6]. Concerning the factor that influences happiness at work, several factors were identified. The first factor is the job characteristics such as salary, promotion, level of danger, and schedule. The second is work characteristics like the environment of the company and the size of the company. The third factor is workers’ characteristics such as gender, age, relationship status, and level of education [7].

Given the significant amount of time spent at work, work happiness is a critical factor in achieving personal well-being and happiness. Fisher [4] indicated that previous studies found that employment has a lower impact on people’s overall happiness than other factors such as a person’s life partner, family leisure, or friends. It does, however, have an established track record of causing unhappiness. Therefore, positive psychology which focuses on the investigation of well-being and happiness as a positive emotion has exploded in the subject of workplace happiness [1]. Workplace happiness is an important issue since most people work for a variety of reasons. It provides a source of income and provides an opportunity to put personal strengths and skills to use, overcome challenges, and attain personal fulfilments [8].

In terms of the measurement of Happiness at Work (HAW), has been the subject of debate amongst scholars around the second decade of the twenty-first century, particularly in the western context [3, 9]. However, the development of the HAW measurement in the eastern context especially in Indonesia still needed to be conducted due to the differences in languages, cultures, and time-lapse. Groundbreaking research in the happiness at work measurement area has been continually conducted. One of the HAW measures was developed by Salas-Vallina, López-Cabrales [9], which was based on Fisher [1]. This existing Happiness at Work Scale has been considered valid and reliable, however, to the best knowledge of the researchers, there is a limitation in terms of the instrument that could be used to assess HAW in the Indonesian context. Therefore, the current study aims to adapt and validate the HAW scale that was developed by Salas-Vallina, López-Cabrales [9] to make it suitable for the Indonesian context.

## Method

### Study design

The current study used a cross-sectional design where all the measures were administered at one point at a time. Ethical approval from University Malaya Research Ethics Committee (UMREC) with the reference number of UM.TNC2/UMREC_1223 had been obtained before the study. The researchers conducted the cross-cultural adaptation by referring to Beaton, Bombardier [10] guidelines, which consist of 6 stages namely, forward translation, synthesis, backward translation, expert committee meetings, pretesting, and submission of the report of the cross-cultural adaptation.

### Participants

The sample of this study consisted of two groups. The first group consisted of 13 experts in psychology and the English language. A total of 11 out of the 13 experts were recruited for the cross-cultural adaptation and the remaining two were recruited for content validity check. Afterward, the 11 experts in charge of cross-cultural adaptation were invited to a focus group discussion to finalize the translation. The second group of this study was lecturers, who were divided into two subsamples. The first subsample consists of 105 lecturers (35 male or 33.3% and 70 female or 66.7%) from a higher education institution in Central Java, Indonesia, which were selected using a convenient sampling method. This sample was used to run exploratory factor analysis (EFA). The second subsample included a total of 391 subjects who are lecturers in private higher education in Yogyakarta, Indonesia, which were selected using a convenient sampling method. Only 370 out of the 391 (147 or 39.73% male and 223 or 60.27% female; mean age = 39.2 years; SD = 9.18 years) were included in the final analysis after removing outliers and incomplete responses. The demographic data of the sample is presented in Table 1.

**Table 1:** Demographic profile of respondents of field study.

Demographic	Level	Frequency	Percentage (%)
**Age**	Below 30 years	65	17.6%
	31–40 years	164	44.3%
	41–50 years	90	24.3%
	Above 50 years	51	13.8%
**Gender**	Male	147	39.73%
	Female	223	60.27%
**Education Background**	Master’s Degree	326	88.1%
	Doctorate Degree	44	11.9%
**Region**	Sleman	129	34.9%
	Bantul	166	44.9%
	Kota	75	20.3%
**Functional Degree**	None	67	18.1%
	Instructor	160	43.2%
	Assistant Professor	121	32.7%
	Associate Professor	21	5.7%
	Professor	1	0.3%

### Measures

In the current study, researchers adapted the HAW questionnaire that was developed by Salas-Vallina, López-Cabrales [9] into the Indonesian context. The scale has 31 items with three components, first, work engagement (WENG), measured using 17 items with a 6-point range from 1 “never” to 6 “always”. The second component is job satisfaction (JS), measured using 6 items with a 5-point ranging from 1 “totally disagree” to 5 “totally agree”. The third component is an affective organizational commitment (AOC), measured using 8 items with a 7-point ranging from 1 “totally disagree” to 7 “totally agree”. Singh and Aggarwal [3] indicated that HAW is a valid and reliable scale with the Cronbach’s Alpha value of 0.991 which indicates high reliability. Some studies that used the HAW Scale also reported good reliability values [e.g., 11, 12].

### Translation

Researchers started the process by first, asking three Indonesian experts in psychology, who have prior knowledge about the concept HAW to do forward translation I. The mother tongue of these experts is the Indonesian language, they were requested to translate the items statements from English into the Indonesian language. In addition, another two experts who are specialists in the English language were recruited to do (forward translator II) by revising the forward translation I also, to compare and detect the different meaning from the original version (English) and highlight the ambiguous meaning from the English version.

In the second stage, both forward translators, with the help of an observer who is a lecturer and a Ph.D. student majoring in English, held a discussion to synthesize the translation result. The resulting version of this second stage is called common translation [10], which is the Indonesian version of HAW (I-HAW). The third stage was backward translation with the help of two translators. In this stage, the translators translated the common translation, which is in the Indonesian language resulted from the second stage, back to English. The fourth stage was holding the expert committee meeting, to facilitate discussion between the researchers with the forward translators, back translators, methodologists, psychology professionals, and language professionals to get the second version of I-HAW.

The fifth stage was pretesting of the second version of I-HAW. This step was done by administering the questionnaire to five subjects who completed the second version of I-HAW. Then, the subjects were interviewed to explore whether the perceptions of the subjects are in line with the meaning of each item in the scale. This is to ensure that the newly developed version is still retaining its similarity in an applied situation [10]. Finally, in the last stage, the committee submitted the reports to the researchers.

### Content validity

After finishing the cultural adaptation, the researchers asked another two experts to assess the content and face validity to calculate the I-CVI and S-CVI. As discussed previously, content validity evidence can be seen from the CVI [13–15]. To assess the content and face validity, researchers followed the recommendation from Yusoff [16]. In the first stage, the researchers prepared a content validation form to ensure the experts’ panel has well-defined expectations and an understanding of what they must do. The researchers provided the names and the definitions of the domain to help the expert in giving scores. They were also provided with the instruments, the objectives of the study, the research questions, the conceptual framework, the definition of terms, and the judgment forms. In the second stage, the researchers chose the two expert panel members based on their expertise related to the topic of the study. According to Yusoff [16], the acceptable CVI value for two experts is at least 0.80. The two experts were asked to evaluate whether the items of the I-HAW are relevant, clear, and essential by rating each item on a 4-point Likert scale (1  =  not relevant, 2  =  somewhat relevant, 3  =  quite relevant, and 4  =  very relevant). Items with a rating of 1 and 2 are considered invalid and items with a rating of 3 and 4 are considered valid. Content validity experts were also requested to review the domains analytically and the items’ representation of the domain prior to providing the score for each item.

### Procedures

The current study was started by conducting a cross-cultural adaptation of the existing scale. The I–HAW scale has also been checked by experts for content validity review. The researchers acquired ethical approval from the University Malaya Research Ethics Committee (UMREC) with the reference number UM.TNC2/UMREC_1223 before the commencement of data collection. The data was collected from big samples for conducting exploratory factor analysis (EFA) and confirmatory factor analysis (CFA). The researchers briefed the respondents about the objectives of the study and required them to sign a consent form if they agree to participate in this study. The I–HAW scale was then distributed to 105 lecturers using an online platform for the pilot study. Due to the spread of the Covid-19 pandemic, the respondents were recruited using convenient sampling. Participation in this study was voluntary. Afterward, the researchers perform the validity and reliability analysis. Furthermore, the data collection for the actual study was carried out with a sample of 391 lecturers in Yogyakarta, Indonesia.

### Data analysis

To analyze the data, the researchers used several methods. Firstly, EFA was used to analyze the dimensionality and the factor loading for the items under each dimension by using IBM SPSS software. Afterward, the researchers conducted CFA for analyzing the field research data using IBM SPSS AMOS software. Prior to performing CFA, researchers conducted data screening and managed missing values, only 370 respondents were included in the CFA analysis.

## Results

The results of this study are presented in three sections namely, content validity results, exploratory factor analysis results, and confirmatory factor analysis results. Prior to that, researchers provided the descriptive analysis results to be further elaborated in the next section.

### Descriptive analysis

The basic descriptive analyses were used to confirm the normal distribution by looking at the pattern and shape of the sample distribution on Happiness at work, and the results of these analyses are provided in Table 2.

**Table 2:** The description of happiness at work construct.

		total_jobsat	total_vigwk	total_abswk	total_aoc
N	Valid	370	370	370	370
	Missing	0	0	0	0
Mean		15.1937	20.5362	15.9676	16.0108
Std. Deviation		2.90344	3.06527	3.15611	3.28335
Skewness		‒0.664	‒0.611	‒0.022	‒0.847
Std. Error of Skewness		0.127	0.127	0.127	0.127
Kurtosis		0.405	0.392	0.042	0.910
Std. Error of Kurtosis		0.253	0.253	0.253	0.253

Note: total_jobsat = total score of job satisfaction component; total_vigwk = total score of vigor at work component; total_abswk = total score of absorption at work component; total_aoc = total score of affective organizational commitment component.

From Table 2, it can be seen that the mean score of the HAW components are as follows: 15.19 (*SD* = 2.90) for job satisfaction component, 20.53 (*SD* = 3.06) for vigor at work component, 15.96 (*SD* = 3.15) for absorption at work component, 16.01 (*SD* = 3.28) for affective organizational commitment component. The Skewness and Kurtosis values are within the acceptable range of normal distribution of the dataset, i.e. score between -2 to +2 [17].

### Content validity results

The last stage of the content validity process calculated the CVI (I-CVI and S-CVI). Before computing CVI, the relevance rating was recorded to a score of 1 for (relevance scale of 3 or 4) or 0 for (relevance scale of 1 or 2). The results are presented in Table 3.

**Table 3:** I-CVI and S-CVI of scale of happiness at work.

Item	Expert 1	Expert 2	Expert Agreement	I-CVI	UA
A1	1	1	2	1	1
A2	1	1	2	1	1
A3	1	1	2	1	1
A4	1	1	2	1	1
A5	1	1	2	1	1
A6	1	1	2	1	1
A7	1	1	2	1	1
A8	1	1	2	1	1
A9	0	1	1	0,5	0
A10	1	1	2	1	1
A11	1	1	2	1	1
A12	1	1	2	1	1
A13	1	1	2	1	1
A14	1	1	2	1	1
A15	1	1	2	1	1
A16	1	1	2	1	1
A17	1	1	2	1	1
A18	1	1	2	1	1
A19	1	1	2	1	1
A20	1	1	2	1	1
A21	1	1	2	1	1
A22	1	1	2	1	1
A23	1	1	2	1	1
A24	1	1	2	1	1
A25	1	1	2	1	1
A26	1	1	2	1	1
A27	1	1	2	1	1
A28	1	1	2	1	1
A29	1	1	2	1	1
A30	1	1	2	1	1
A31	1	1	2	1	1
**Proportion relevance**	**0,97**	**1,00**	**S-CVI/Ave**	**0,98**	
**The average proportion of items**			**S-CVI/UA**		**0,97**

As shown in Table 3, it can be concluded that almost all the I-CVI scores of items on the I-HAW scale are 1, which means the items meet a satisfactory level of I-CVI of more than 0.80. Except for item A9 that has a score of 0.5 for the I-CVI level. Therefore, the researchers have amended item A9 to improve its quality. Furthermore, the score of S-CVI was 0.98 meaning that the scale also meets the satisfactory level of S-CVI of more than 0.80. The final version of I-HAW contains 31 items distributed under 3 dimensions.

### Correlation analysis

The coefficient of reliability of HAW constructs namely job satisfaction, vigor at work, absorption at work, and affective organization commitment, are showed in Table 4 below.

**Table 4:** Correlation analysis.

Correlations					
		total_jobsat	total_vigwk	total_abswk	total_aoc
total_jobsat	Pearson Correlation	1	.600**	0.243**	0.657**
	Sig. (2-tailed)		0.000	0.000	0.000
	N	370	370	370	370
total_vigwk	Pearson Correlation	0.600**	1	0.329**	0.655**
	Sig. (2-tailed)	0.000		0.000	0.000
	N	370	370	370	370
total_abswk	Pearson Correlation	0.243**	0.329**	1	0.331**
	Sig. (2-tailed)	0.000	0.000		0.000
	N	370	370	370	370
total_aoc	Pearson Correlation	0.657**	0.655**	0.331**	1
	Sig. (2-tailed)	0.000	0.000	0.000	
	N	370	370	370	370

Note: total_jobsat = total score of job satisfaction component; total_vigwk = total score of vigor at work component; total_abswk = total score of absorption at work component; total_aoc = total score of affective organizational commitment component.

Based on the analysis, it can be inferred that intercorrelation of the sub-construct is significant. As it is seen that the correlation between vigor at work and job satisfaction is 0.600; the correlation between vigor at work and absorption at work is 0.329, the correlation between job satisfaction and absorption at work is 0.243; the correlation between affective organizational commitment and job satisfaction is 0.657; and the correlation between absorption at work and affective organizational commitment is 0.331. All the correlation coefficient results are significant at *p* = 0.01.

### Exploratory factor analysis results

This stage aimed to measure the dimensionality of the I-HAW Scale. According to Hair, Black [17], researchers employed EFA for several reasons. Firstly, EFA can be used to determine the usefulness or suitability of items through factor loading and their dimensionality. Secondly, EFA analyzes the relationships among items in its most common arrangement by describing the underlying dimensions. Thirdly, EFA can be employed to explore and assess the instruments in terms of some factors such as culture, languages, time-lapse, and study subjects. Aligned with Hair, Black [17], EFA is needed to get validity, reliability, and decent measurement [18–20]. Finally, the EFA algorithm can be implemented in the newly modified items to reestablish validity and reliability [21, 22].

EFA was conducted using SPSS software. Varimax rotation with eigenvalues greater than 1 was employed for the rotation. The results indicated that the Keizer-Meyer-Olkin (KMO) sample adequacy test was 0.807 and significant at *p* > 0.05. Meaning that the sample is adequate to run EFA, see Table 5. The KMO score is applied to measure the data factorability and to guarantee sampling adequacy. If the value of sampling adequacy is sufficient, then the factor analysis can be conducted [23]. The KMO value that is more than 0.6 is considered outstanding [24]. Since the results of Bartlett’s Test KMO showed that the data is sufficient to continue with the next step, researchers proceeded to the data reduction procedure in EFA [24].

**Table 5:** Kaiser–Meyer–Olkin and Bartlett’s sphericity tests.

Kaiser-Meyer-Olkin Measure of Sampling Adequacy.		.807
Bartlett’s Test of Sphericity	Approx. Chi-Square	2201.063
	df	465
	Sig.	0.000

The EFA results revealed that around six factors explain 66.331% of the HAW in the Indonesian context, see Table 6. According to Hair, Black [17], total variance of 60% or even less than 60% is considered acceptable for social sciences. Meaning that these factors and items are adequate to measure the construct of HAW. In other words, 66% of happiness at work construct was attributed to or explained by the six factors and the remaining might be due to other factors.

**Table 6:** Total variance explained.

Component	Initial Eigenvalues			Rotation Sums of Squared Loadings		
	Total	% Of Variance	Cumulative %	Total	% Of Variance	Cumulative %
1	11.584	37.369	**37.369**	4.670	15.064	15.064
2	2.851	9.198	46.567	3.702	11.941	27.005
3	2.001	6.454	53.021	3.522	11.362	38.367
4	1.591	5.133	58.154	3.288	10.605	48.972
5	1.401	4.520	62.674	2.701	8.714	57.686
6	1.134	3.657	**66.331**	2.680	8.645	**66.331**

Extraction Method: Principal Component Analysis.

In the second round of EFA analysis, researchers run the analysis by suppressing a small coefficient to 0.45 as recommended by [24]. Based on the results of this round of analysis, 11 items were deleted due to low factor loading and cross-loadings. The rest of the items are presented in Table 7.

**Table 7:** The item and component of work happiness.

Component	No. of Items	Items	Reliability
1	4	A28, A29, A30, A31	0.935
2	5	A1, A2, A8, A9, A13	0.852
3	1	A12	-
4	4	A19, A20, A22, A23	0.770
5	5	A6, A11, A14, A25, A27	0.749
6	1	A10	-
**All items**	**20**	**20**	

As shown in Table 7, factors 3 and 6 have only one item loaded under each factor. Therefore, these two factors were deleted. The prefinal version of I-HAW that was prepared for CFA included four factors and 18 items, see Table 8. The first component consists of 4 items, the second consists of 5 items, the third component consists of 5 items, and the last component consists of 4 items.

**Table 8:** The components and their respective items.

Rotated Component Matrix				
	Component			
	1	2	3	4
A19	.743			
A20	.694			
A22	.697			
A23	.710			
A1		.613		
A2		.744		
A8		.766		
A9		.756		
A13		.612		
A6			.665	
A11			.643	
A14			.637	
A25			.600	
A27			.610	
A28				.787
A29				.851
A30				.823
A31				.807

Extraction Method: Principal Component Analysis.

Rotation Method: Varimax with Kaiser Normalization.

a. Rotation converged in 6 iterations.

In the following step, researchers tested the reliability of the I-HAW and the result is presented in Table 9.

**Table 9:** Internal reliability for the construct.

Component	N of items	Items	Cronbach’s Alpha for each sub-factor
1	4	A19, A20, A22, A23	0.770
2	5	A1, A2, A8, A9, A13	0.852
3	5	A6, A11, A14, A25, A27	0.749
4	4	A28, A29, A30, A31	0.935
**Total**	**18**	**18**	**0.887**

### Confirmatory factor analysis results

**Fig 1:**
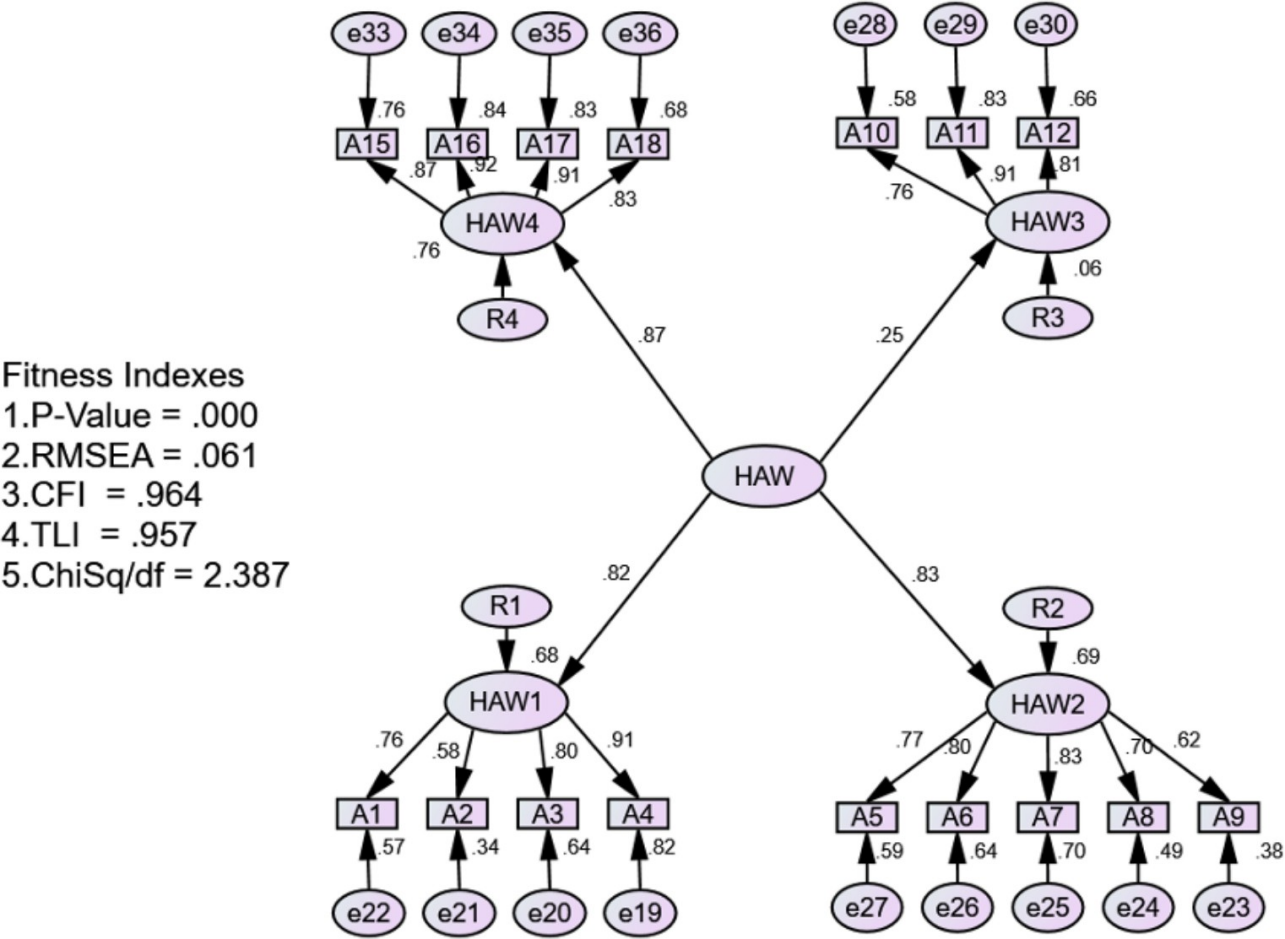
The result of confirmatory factor analysis.

The confirmatory factor analysis (CFA) was performed on the 18 items of the I-HAW Scale resulting from the EFA. CFA also serves to assess three aspects including unidimensionality, validity, and reliability [25, 26]. The unidimensionality assessment was carried out before measuring validity and reliability. Unidimensionality will only be obtained if the item factor loading score is > 0.6. Thus, to obtain unidimensionality from the measurement model, items with a low factor loading score must be eliminated [24]. The results of CFA are presented in Fig 1.

Fig 1 showed the fit indices for the second-order factor of the HAW construct in the Indonesian context. Since the fit indices of the measurement were met, we can declare that the validity and reliability of the I-HAW are achieved [17]. Construct validity was also assessed using convergent and discriminant validity, which are types of validity that the measurement model of latent constructs must be passed through [17]. Based on the analysis, all the items loaded significantly at a *p* value of < 0.05. However, the two items under component 3 (items A13 and A14) were deleted due to low factor loading. All the fit indices met the cut-off scores, and the results are presented in Table 10.

**Table 10:** Construct validity assessment.

Category	Index	Acceptance Level	Index Value	Result
Absolute Fit	RMSEA	< 0.08	0.61	The required level is achieved
Incremental Fit	CFI	> 0.9	0.964	The required level is achieved
Parsimonious Fit	Chisq/df	< 5.0	2.389	The required level is achieved

It can be seen from the fitness indexes that the measurement model of the HAW has met the criteria for construct validity. After measuring the construct validity, the Average Variance Extracted (AVE) was measured to test the convergent validity and discriminant validity. Meanwhile, composite reliability (CR) was assessed to test the reliability of the measurement model. The results revealed that the convergent validity of the HAW construct is achieved since all the scores of AVE are > 0.5. Furthermore, the composite reliability is also satisfactory, as all the scores of CR are > 0.6 [27]. Table 11 summarizes the results of AVE and CR.

**Table 11:** The results of AVE and CR.

Construct	Item	Factor Loading	CR (Above 0.6)	AVE (Above 0.5)	Convergent Validity
HAW 1	A1	0.75	0.845	0.583	Ok
	A2	0.58			
	A3	0.79			
	A4	0.90			
HAW 2	A5	0.80	0.884	0.606	Ok
	A6	0.81			
	A7	0.84			
	A8	0.76			
	A9	0.67			
HAW 3	A10	0.76	0.867	0.687	Ok
	A11	0.90			
	A12	0.82			
	A13	Items were deleted because of low factor loading			
	A14				
HAW 4	A15	0.86	0.932	0.778	Ok
	A16	0.92			
	A17	0.91			
	A18	0.83			

**Fig 2:**
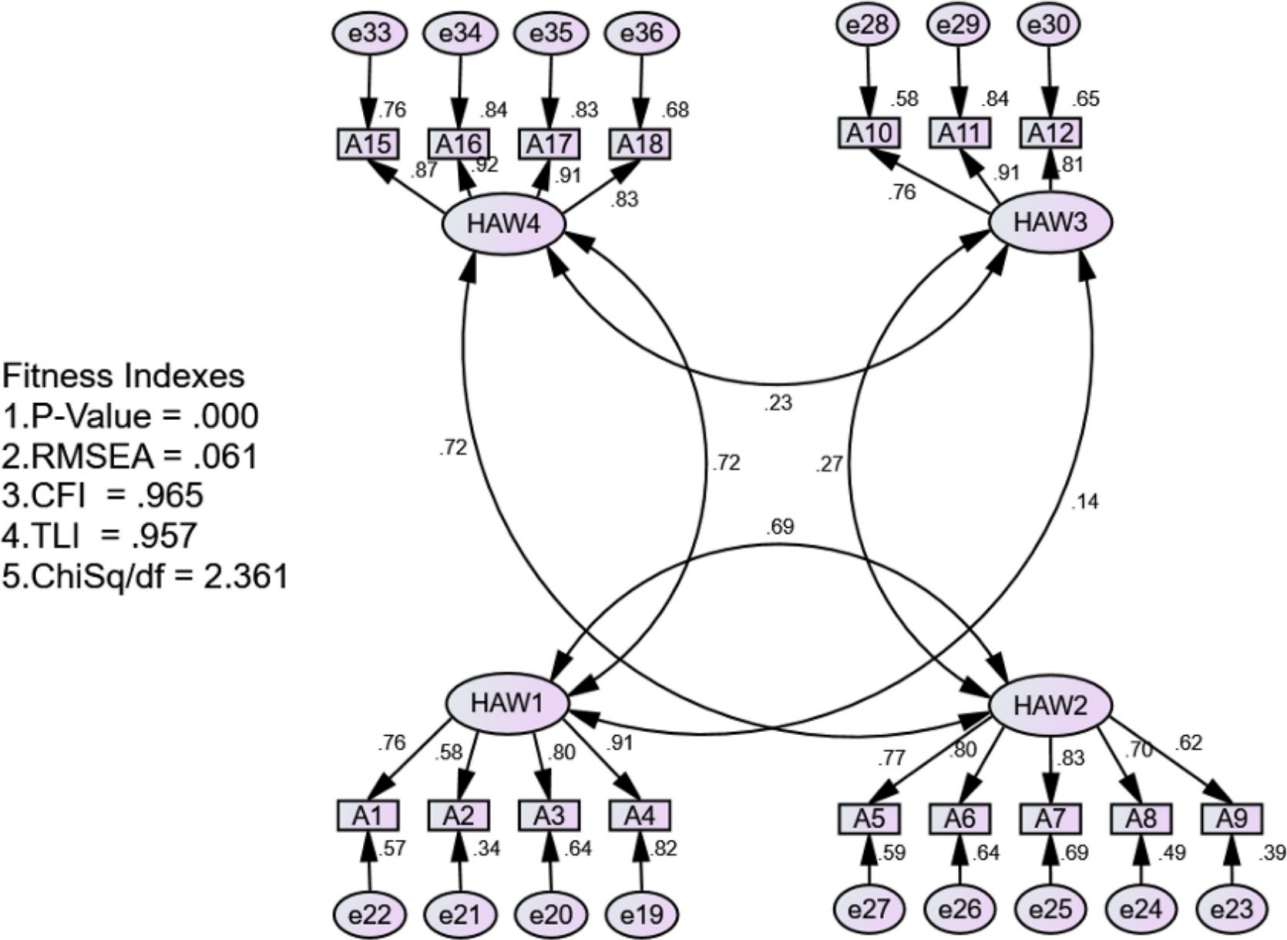
The results of discriminant validity for HAW.

Based on the result shown in Table 11 above, the convergent validity has met the criteria indicated by the acceptable value of Average Variance Extracted (AVE), which is more than 0.5. In addition, the criterion of the composite reliability (CR) has also fulfilled the acceptable value, which is more than 0.6. The correlation coefficient between all four components of the model to measure discriminant validity was calculated using IBM-SPSS-AMOS and presented in Fig 2.

Based on the analysis, it was found that the correlation coefficients between the factors are 0.23, 0.27, 0.69, 0.72, 0.14, and 0.72. To conclude, it is seen that the correlation coefficient of the six components does not exceed the maximum value of 0.85, which means that the HAW has good discriminant validity.

### Reliability of I-HAW

The final stage of evaluating the psychometric properties of I-HAW was assessing the reliability using Cronbach alpha. Cronbach’s alpha can be used to measure the reliability. It ranges from 0 to 1. The values of 0.60 to 0.70 considered the lower limit of acceptability [17]. The results indicated that all the sub-factors have good reliability ranging from (0.843–0.931), see Table 12. While the Cronbach alpha for the whole scale is 0.946, meaning that I-HAW has a good reliability.

**Table 12:** Reliability analysis for I-HAW sub-factors.

Variables	Cronbach alpha
job satisfaction	0.843
vigor at work	0.861
absorption at work	0.866
affective organizational commitment	0.931

## Discussion

The main objective of this study was to adapt the HAW questionnaire to the Indonesian context. This study is essential because it is rare to find a scale to measure HAW in the Indonesian context. Therefore, the main contribution of the current study is that it provides a tool that can help researchers to study and assess happiness at work in a non-western context, especially in Indonesia. Researchers conducted cultural adaptation by applying forward and back-to-back translation with the supports of some experts. The cross-cultural adaptation was done due to the different language and the culture of the original version of the instrument, which cannot be used as it is in the Indonesian context without cultural adaption. The result of the cross-cultural adaptation meets the requirement of semantic, idiomatic, experiential, and conceptual equivalence. The meaning and wording of the resulting version of HAW are similar to the original one. It was also found that the original and the target version of the HAW Scale has experiential equivalence. Thus, it can be demonstrated that the cross-cultural adaptation has resulted in the Indonesian version of the HAW (I-HAW) scale which is equivalent to the original scale.

The second draft of I-HAW was administered to a big sample to test the dimensionality of the scale and it was found that six factors explained more than 60% of the variance. However, researchers deleted two factors due to the lack of items loaded under these two factors. The researchers also deleted 11 items due to the low factor loading. Unlike the original version from Salas-Vallina, López-Cabrales [9], the result of the EFA produced I-HAW with four factors and 20 items. This could be attributed to the cultural differences and the collectivist culture of the Indonesians, which might influence their point of view about HAW. As indicated by past studies, one of the drivers of happiness is culture. It has been stated that culture has a considerable impact on happiness [28]. Furthermore, various cultures will have varying levels of happiness and different predictors of happiness [29]. In an individualist society, for example, emotion is a powerful predictor of life satisfaction. In a collectivist society, on the other hand, the norm plays a critical role in predicting life satisfaction. It’s also possible that different civilizations have distinct ways of answering inquiries [30]. To summarize, individualists and collectivists have differing perspectives on happiness.

However, the results of this study are in line with the results of Singh and Aggarwal [3], who identified four factors. The four EFA factors are latent variables from which items have been designed to originate. On the surface, however, some variables appear to be a formative measure of happiness at work. On the other hand, another study conducted in Spain by Ramirez-Garcia, Perea [8] reported that the EFA results indicated that only two factors explained the major variance in HAW.

After extracting the factor in EFA analysis, researchers proceed to the next step which is confirming that the extracted factors measure a single construct. The result of CFA leads to a new version of I-HAW that consists of four components with 16 items. The four components measure job satisfaction, vigor at work, absorption at work, and affective organizational commitment. All the goodness of fit indices met the cut-off scores recommended by Hair, Black [17]. The results also indicated that I-HAW have high reliability, which is in line with previous studies [3, 9].

I-HAW supports former studies on positive attitudes and adopts the definition of HAW from Fisher [1] which consist of three dimensions that broadly capture HAW, including affective implications and feelings at work, evaluative judgments of job characteristics like salary, supervision, and career opportunities, and feelings of belonging to the organization. All these dimensions or aspects of the definition are reflected in the I-HAW domains. The current study has some implications for employees and other researchers. First, I-HAW aims to inspire companies in Indonesia to entice businesses to create better working conditions. Employees are projected to become more involved, contented, and devoted at work as a result of this, which aligns with recent publications focused on happiness and the common good [31]. Second, HAW is a strong tool that may assist businesses in attracting the kind of innovative, eager, and passionate individuals who help them succeed. Thus, HAW should become a key emphasis of human resource management, and its accurate measurement is a must-have technique [32].

Third, this study can help other researchers interested in investigating happiness at work in the Indonesian context by providing them with a contextualized tool that can facilitate the assessment of HAW among Indonesian. The I-HAW scale can be useful also for Indonesian lecturers, it will help to know the level of lecturer’s happiness at work, which can help the lecturers to know the determinants of the work happiness itself and finally can increase its level. A high level of happiness leads to a high level of performance.

In addition, the current study is in line with the recommendations of [32] who suggested the considerations of cultural diversity as cultural norms vary between the countries which is proven by the results of the current study. The result of this research also adds to the literature of HAW by giving a new version of HAW that is valid and reliable to be used by future studies in non-western contexts specifically in Indonesia. However, researchers recommend future studies do more comparative cultural research with HAW for the lecturer’s version scale. Therefore, this research can specifically contribute to the field of measurement of HAW in the Indonesian context. Future research can add other approaches such as interviews and focus group discussion in investigating HAW. Other researchers who want to study work happiness in the Indonesian context can refer to the finding of this study as a starting point.

## Conclusion

The result of this study concluded that different contexts might have different dimensionality of a particular construct. It happens to the Indonesian context that has different dimensionality on HAW measurement that could be attributed to the different perspectives about happiness in different cultures. In the case of Indonesia, it belongs to Eastern countries that stick to collectivist culture, and this kind of culture might see happiness differently. The findings of this study indicated that I-HAW has high reliability and good validity. It can be concluded that I-HAW consists of 16 items and four distinct factors that demonstrated significant distinctions to deserve considering them as separate and unique variables. This study can help other researchers interested in researching work happiness in the Indonesian context.

## Limitations

Prior data collection researchers were planning to select the samples using a random sampling method and use a hard copy questionnaire. However, due to pandemic Covid-19, the researchers were not allowed to give the hard copy questionnaire directly to the research respondents and the questionnaire was distributed online using google form. Using an online survey gives the limitation to the study which makes the sampling method become convenience sampling.

## Supporting information

S1 Data EFA analysis data. (XLSX)

S2 Data Field study data. (XLSX)
